# Case Report: Anti-NMDAR Encephalitis With Anti-MOG CNS Demyelination After Recurrent CNS Demyelination

**DOI:** 10.3389/fneur.2021.639265

**Published:** 2021-02-24

**Authors:** Bing-Yan Ren, Yi Guo, Jing Han, Qian Wang, Zai-Wang Li

**Affiliations:** ^1^Department of Emergency, Nantong First People's Hospital, The Second Affiliated Hospital of Nantong University, Nantong, China; ^2^Department of Neurology, Shenzhen People's Hospital, The Second Clinical Medical College of Jinan University, The First Affiliated Hospital of Southern University of Science and Technology, Shenzhen, China

**Keywords:** recurrent CNS demyelination, diagnosis, anti-NMDAR encephalitis, anti-MOG CNS demyelination, therapy

## Abstract

**Introduction:** Anti-*N*-methyl-d-aspartate receptor (NMDAR) encephalitis, a serious neurological autoimmune disorder caused by autoantibodies with diverse clinical manifestations, may simultaneously onset with antimyelin oligodendrocyte glycoprotein (MOG) demyelination after recurrent central nervous system (CNS) demyelination.

**Case Report:** We present a case of anti-NMDAR encephalitis combining with anti-MOG CNS demyelination following recurrent CNS demyelination. A 38-year-old man admitted to hospital developed epileptic seizures following recurrent episodes of cross-sensory disturbance and dizziness. Magnetic resonance imaging (MRI) showed a demyelinating lesion in the right brainstem initially. Despite a good response to methylprednisolone pulse therapy at the beginning, the patient still had relapses and progression after corticosteroid reduction or withdrawal. Then brain MRI discovered new serpentine lesions involving extensive cerebral cortex on his second relapse. Repeat autoantibodies test indicated cerebrospinal fluid (CSF) NMDAR antibodies coexisted with MOG-Abs simultaneously, suggesting the diagnosis of anti-NMDAR encephalitis with anti-MOG CNS demyelination.

**Results:** After a definite diagnosis, the patient was treated with mycophenolate mofetil (MMF) and corticosteroid. He was discharged after his symptoms ameliorated. No neurological sequels remained, and there were no effects on his activities of daily living after 6 months of immunoregulatory therapy of MMF and corticosteroid.

**Conclusion:** For individuals with recurrent CNS demyelination, especially combining with cortical encephalitis, repeated detection of autoantibodies against AE, and demyelination in CSF/serum can be helpful to enable a definite early diagnosis. For patients who suffer from anti-NMDAR encephalitis combining with anti-MOG CNS demyelination, second-line immunotherapy is recommended when first-line treatment such as steroids, intravenous immunoglobulin G (IVIG) and plasma exchange has been proven ineffective to prevent the relapse of disease.

## Introduction

Anti-*N*-methyl-d-aspartate receptor (NMDAR) encephalitis is an autoimmune disorder in association with immunoglobulin G (IgG) antibodies against the GluN1 subunit of the NMDAR (1) and clinical manifestations are diverse such as psychiatric behavior or cognitive dysfunction, speech dysfunction, seizures, dyskinesias, loss of consciousness, autonomic dysfunction, or central hypoventilation (2). Despite severe symptoms, only 35% of cases reveal abnormal brain MRI at the onset involving cortical and subcortical regions of the brain, hippocampus, and sometimes the basal ganglia, which are often mild, transient, and nonspecific (3). As few case reports of anti-NMDAR encephalitis combining with demyelinating disorders such as acute demyelinating encephalomyelitis (ADEM), myelitis, or neuromyelitis optica (NMO) in recent years (4), patients suffering from anti-NMDAR encephalitis and a demyelinating disorder concurrently or successively may be easily misdiagnosed. A case report described MOG-antibody-mediated recurrent demyelination following autoimmune cortical encephalitis in 2017 (5). However, the case of anti-NMDAR autoimmune cortical encephalitis after MOG-antibody-mediated recurrent demyelination was relatively rare as far as we know.

Here, we describe a case of anti-NMDAR encephalitis with anti-MOG CNS demyelination following recurrent CNS demyelination according to neuroimaging characteristics, laboratory test, neuroelectrophysiological examination and autoantibodies test in cerebrospinal fluid (CSF)/serum.

## Case Presentation

A previously healthy 38-year-old man presented to the local hospital with progressive right facial numbness and left hemianesthesia (below the left inferior cervical level), accompanied by diplopia, blurred vision, vertigo, gait unsteadiness, nausea, and vomiting. His brain MRI on 14th March 2018 showed a lesion in the right brainstem (hypointensity on T1WI but hyperintensity on T2WI, FLAIR, DWI, ADC, and ring enhancement around the lesion (Supplementary Figure 1). He was preliminarily diagnosed with probable CNS demyelination and empirically treated with high-dose methylprednisolone. His clinical symptoms were markedly improved within 2 weeks, and he was discharged with an oral prednisone taper. However, 1 week after the discontinuation of oral prednisone, his symptoms reappeared soon. The patient denied history of hypertension, diabetes, or any other high-risk factors for cerebral vascular diseases (CVD). A comprehensive neurological examination revealed limited right ocular abduction with horizontal nystagmus, right-sided central facial paralysis, right facial hypoesthesia, hemihypesthesia below the left inferior cervical vertebrae, and a left suspect Babinski sign without meningeal irritation sign or abnormal reflexes.

**Figure 1:**
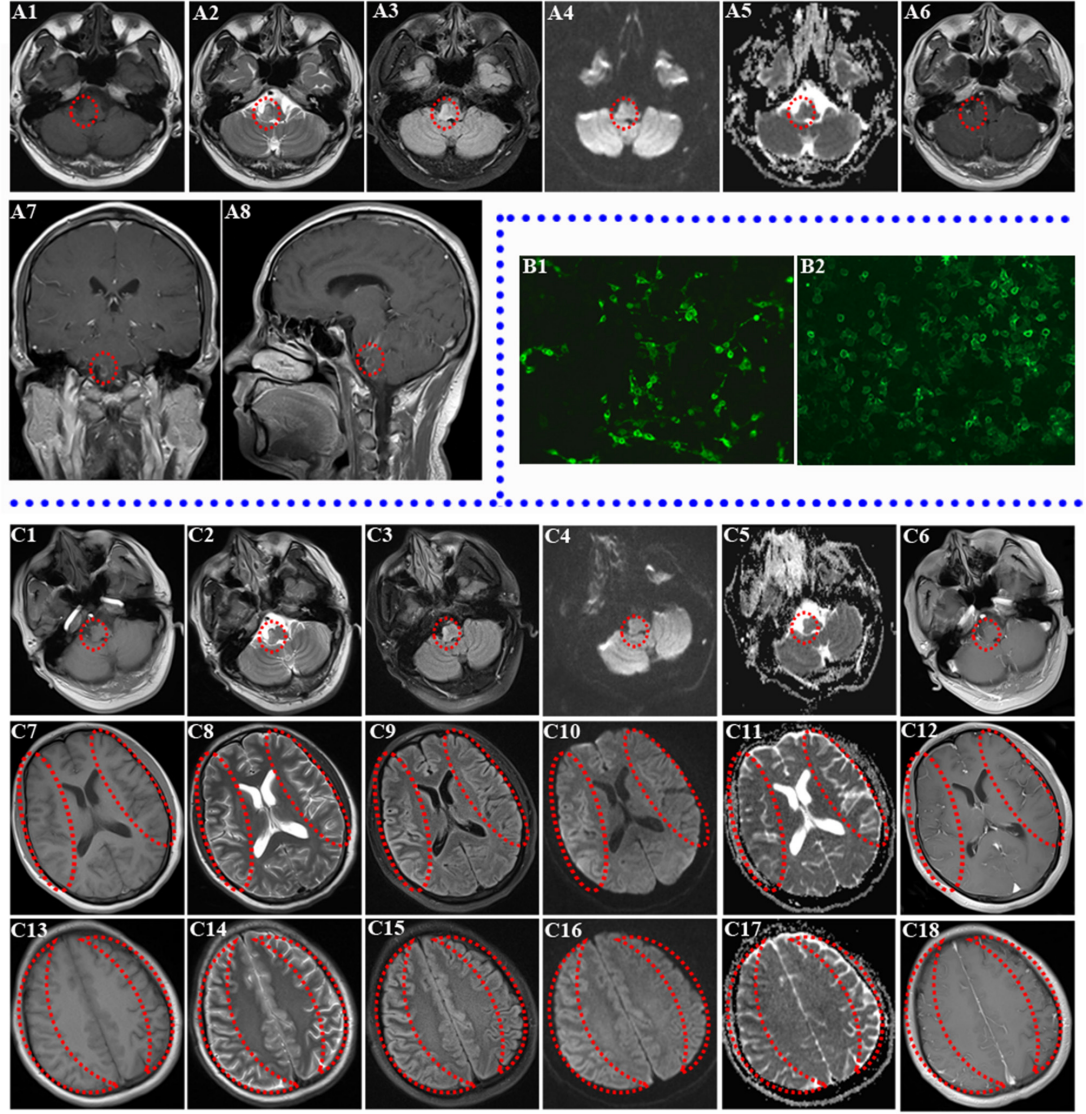
Brain MRI of two relapses and the result of re-examined autoantibodies. **(A1–A8)** Brain MRI of first relapse (abnormal signal in right medulla oblongata and pontine; the lesions are marked by a red circle). Decreased T1 signal abnormalities **(A1)** and increased T2 **(A2)**, FLAIR **(A3)**, DWI **(A4)**, and ADC **(A5)** signal abnormalities involving the right brainstem are shown. Ring Gd DTPA enhancement around the lesion still existed in the brainstem in axial review **(A6)**, sagittal review **(A7)**, and coronal review **(A8)** in brain MRI of first relapse. **(B1–B2)** Transfected, cell-based indirect immunofluorescence test showed positive anti-NMDAR in CSF and anti-MOG antibodies in serum of patient. **(B1)** The patient's CSF was positive for anti-NMDAR antibodies. **(B2)** The patient's serum was positive for anti-MOG antibodies. **(C1–C18)** Brain MRI of second relapse (abnormal signals in the right brainstem, diffuse abnormal signals in the cerebral cortex of both hemispheres, the obvious lesions are marked by red circle and crescent-shaped). Decreased T1 **(C1)** signal abnormalities and increased T2 **(C2)**, FLAIR **(C3)**, DWI **(C4)**, and ADC **(C5)** signal abnormalities still existed in the right brainstem without obvious Gd DTPA enhancement **(C6)**. Decreased T1 **(C7)**, ADC **(C11)** signal abnormalities, and increased T2 **(C8)**, FLAIR **(C9)**, and DWI **(C10)** signal abnormalities involving both cortexes at the basal ganglia level without obvious Gd DTPA enhancement **(C12)** are shown. Right cortex serpent-like lesion is more obvious. Decreased T1 **(C13)** and ADC **(C17)** signal abnormalities and increased T2 **(C14)**, FLAIR **(C15)**, and DWI **(C16)** signal abnormalities involving both cortexes at the centrum semioval level without obvious Gd DTPA enhancement **(C18)** are shown (right cortex serpent-like lesion is more obvious).

Given the patient's myriad of symptoms, we concluded a right medulla oblongata and pons lesion involving the spinal trigeminal nucleus, lateral spinothalamic tract, facial nucleus, vestibular nucleus, and abducens nucleus, which was later confirmed upon a brain MRI scan on 5th May 2018 (Figures 1A1–A8). Consequently, in order to determine whether the patient suffered from CNS demyelination and ensure him to achieve effective treatment in time, lumbar puncture was provided immediately upon admission on the same day. CSF analysis revealed leukocytosis (88 × 106/L; 90% lymphocytes), mild increased protein (0.48 g/L), normal glucose (4.35 mmol/L), and chlorine (119.8 mmol/L), suggestive of mild inflammatory reaction. There were no bacterial, fungal, acid-fast bacilli, cryptococci, or viral antibodies in CSF, and oligoclonal bands (OCB) were negative. In addition, the report on 8th May 2018 revealed that autoantibodies against AE and demyelination diseases were negative in CSF/serum. Despite insufficient diagnostic evidence, he was still considered to have CNS demyelination. In addition, immunoregulatory therapy (methylprednisolone, 500 mg/day intravenously for 5 days with an oral prednisone taper and intravenous IgG, 0.4 g/kg/day for 5 days) were applied empirically at the same time. He responded well to the treatment and then was discharged with residual mild blurred vision 2 weeks after this relapse.

Unfortunately, the patient was readmitted a month and a half after this discharge due to left facial and limb epileptic seizures, followed by a generalized tonic-clonic seizure with loss of consciousness for 2 min. He had weakness in his left limbs after consciousness recovery. Upon admission, he additionally presented with somnolence, dysarthria, shallower left sulci nasolabialis, left skew of tongue, cross sensory disturbance (hypalgesia in right face and left side below the inferior cervical vertebrae), left limb hemiplegia (muscle strength grade 4), active left tendon reflex, and a left Babinski sign. Follow-up brain MRI on 4th July 2018 discovered new serpentine lesions involving the bilateral frontal, temporal, parietal, and occipital cortex (Lace sign), as well as the original brainstem lesion (Figures 1C1–C18). In addition, laboratory tests revealed increased lactate dehydrogenase (LDH), creatine kinase (CK), positive antinuclear antibody (1:80), and weak positivity for anti-SSA/SSB antibody. However, later labial salivary gland biopsy showed negative result. In addition, mitochondrial encephalomyopathy was excluded due to the absence of mitochondrial gene mutations. With the evolution of the disease, his clinical symptoms became more serious and complex.

Then a second autoantibody test of the aforementioned proteins in the serum/CSF was performed for the purpose of a definitive diagnosis and effective treatment on 10th July 2018. Repeat CSF analysis indicated 275 mmH_2_O IP, leukocytosis (50 × 10^6^/L; 85% lymphocytes), mild increased protein (0.49 g/L), slightly decreased chlorine (126.0 mmol/L), and normal glucose (4.30 mmol/L). Notably, serum anti-NMDAR and MOG antibodies were both positive except autoantibodies against paraneoplastic syndrome. It is interesting that CSF NMDAR antibodies also coexisted with MOG-Abs simultaneously upon retesting (Figures 1B1,B2). Then a diagnosis of anti-NMDAR encephalitis combining with anti-MOG CNS demyelination was confirmed due to the coexistence of both antibodies.

The patient was started on corticosteroid impulse therapy followed by oral tapering with mycophenolate mofetil (MMF) (2,000 mg/day). Six months after the second relapse, he had a good prognosis without any neurological sequels, and there were no effects on his activities of daily living. Surprisingly, the re-examination brain MRI on 9th January 2019 revealed that widespread abnormal signals in the bilateral cerebral cortex and brainstem had almost disappeared after MMF and corticosteroid treatment for 6 months (Supplemental Figure 2).

## Discussion

A case of anti-NMDAR encephalitis combining with anti-MOG CNS demyelination after recurrent CNS demyelination was eventually identified. During the patient's first hospitalization, brain MRI showed a lesion in the right brainstem. Based on the patient's clinical manifestations and neuroimaging characteristics at the first relapse, CVD, CNS infectious diseases, an intracranial mass lesion and demyelinating diseases should be taken into consideration as a cause. Due to the absence of high-risk stroke factors and a vascular injury pathological basis, ischemic infarction should be excluded first. Intracranial infectious diseases can also be ruled out in consideration of his symptoms without headache, fever, meningeal irritation signs, and the characteristics of his CSF test and brain MRI. For negative findings of magnetic resonance spectrum, good response to methylprednisolone therapy and his nonprogressive course, the diagnosis of intracranial mass lesion could be ruled out. Therefore, the patient was preliminarily diagnosed with CNS demyelination according to his brainstem lesions, the relapsing-remitting course, abnormal SEP, BAEP, effective immunotherapy, and neuroimaging characteristics consistent with CNS demyelination.

However, the disease not only relapsed but also progressed (epileptic seizures occurrence and new serpentine lesions involving the bilateral frontal, temporal, parietal, and occipital cortex in brain MRI) during the process of prednisolone reduction after second discharge. Diagnostic confusion arose as the extensive cortex lesions and brainstem lesions seemed not to be explained by a single disease. There has been increasing awareness of CNS demyelination presenting concurrently with other autoimmune diseases, including Sjögren's syndrome, myasthenia gravis (MG), systemic lupus erythematosus (SLE), and sarcoidosis (6). It is reasonable to believe that the patient's condition was related to Sjögren's syndrome due to weak positivity for anti-SSA/SSB antibody in serum. However, Sjogren's syndrome was ruled out because of negative labial salivary gland biopsy results. MG, SLE, and sarcoidosis were also excluded as there was no relative clinical evidence.

In consideration of the diffuse cortical serpentine lesions (lace sign), differential diagnoses of mitochondrial encephalomyopathy, Creutzfeldt-Jakob disease (CJD), hypoxic-ischemic encephalopathy, hypoglycemic encephalopathy, epilepsy-mediated brain damage, encephalitis (including viral encephalitis, immune-related encephalitis, autoimmune encephalitis, limbic encephalitis, etc.), as well as others should be taken into consideration. For the diffuse cortical serpentine lesions (Lace sign) in cranial MRI, differential diagnosis of mitochondrial encephalomyopathy should be taken into consideration. However, the diagnosis was untenable, for lack of the evidence of mitochondrial gene mutations. He had no typical clinical manifestations of CJD (such as progressive dementia, myoclonus), so CJD was not considered. Hypoxic-ischemic encephalopathy and hypoglycemic encephalopathy were also excluded because he had no any relative pathogenesis. The patient did have epileptic seizures, however, the epileptic seizures have not lasted more than 2 min, excluding the diagnosis of epilepsy-mediated brain damage. At the same time, all aforementioned diseases (except encephalitis) do not exhibit such characteristics in brain MRI (diffuse cortical lesions with accompanying brainstem demyelinating lesions).

According to the above clinical analysis, we considered the case to have CNS demyelination or cortical encephalitis due to certain autoimmune mechanism although autoantibodies of AE and myelin in CSF/serum were all negative in the first test. In order to rule out a negative response time window of autoantibody production and the possible influence of immunotherapeutic agents in the detection of autoantibodies, autoantibodies of AE, and myelin were re-examined. Afterwards, the positive CSF NMDAR antibodies and MOG-Abs in the second test prompted us to the diagnosis of anti-NMDAR encephalitis with anti-MOG CNS demyelination.

Anti-NMDAR encephalitis may show lesions of demyelination in some individuals. In a recent investigation, 3.3% of 691 patients with anti-NMDAR encephalitis had clinical or imaging evidence of a demyelinating disorder (4). Acute demyelinating encephalomyelitis (ADEM), neuromyelitis optica (NMO), optic neuritis, myelitis, multiple sclerosis (MS), prominent brainstem dysfunction, and other demyelinating disorders with anti-NMDAR encephalitis have also been reported (7). It also reported that patients with MOG-Abs alone can induce cerebral cortical encephalitis and CNS demyelination at the same time (2). Moreover, a case reported MOG-antibody-mediated recurrent demyelination following anti-NMDAR encephalitis in 2017 (5). However, the case of anti-NMDAR autoimmune cortical encephalitis simultaneously onset with anti-MOG CNS demyelination after recurrent CNS demyelination has not been reported previously as far as we know.

As mentioned earlier, simultaneous appearance of both antibodies in the case may not be accidental but may be caused by some relevance of the immunological mechanisms perhaps. We guessed that mechanism of CNS demyelination may overlap with that of anti-NMDAR encephalitis. Considering that oligodendrocytes do contain NMDAR, it is reasonable to speculate that the immune attack targeting myelin may simultaneously involve NMDAR, and vice versa (5). As we know, differentiation of oligodendrocyte precursor cells (OPCs) into myelinating oligodendrocytes is the most important event for CNS axonal myelination during development and remyelination in demyelinating diseases. A research in 2012 showed that NMDAR plays a critical role in the regulation of promoting OPC differentiation and remyelination by enhancing myelin protein expression through mTOR-dependent mechanism (8). While the anti-NMDAR antibodies target molecules involved in neurotransmission, they block ion channel pores or disrupt the interaction with neighboring molecules and reduce expression of cell surface receptors by altering receptor localization on the membrane or causing receptor internalization which lead to neuronal dysfunction (9). Therefore, the NMDAR is a double-edged sword in patients with demyelinating disorders because it is not only involved in the regeneration and repair of myelin but also associates with autoimmune response leading to a worse prognosis finally.

It was reported in 2020 that 10 children with AE associated with MOG-Abs presented several radiologic features in position: involvement of cortical and deep gray matter structures often associated with juxtacortical signal changes and no involvement of the optic nerves or other white matter structures. In addition, all of them had a brief prodromal period, associated often with severe headache, followed by impairment of consciousness, other neurologic symptoms, and seizures (10), which was consistent with our case. Hence, it suggested the important auxiliary role of imaging features and clinical manifestations in diagnosis. A recent study found that between 4% of patients with anti-NMDAR encephalitis have concurrent glial-Abs including MOG, glial fibrillary acidic protein (GFAP), and aquaporin 4 (AQP4), and people with concurrent glial-Ab often have more frequent previous episodes of encephalitis or demyelinating disorders, more frequent prodromal symptoms, and shorter duration of symptoms (11). Therefore, for patients with recurrent demyelinating disorders and various prodromal symptoms, the examination of immune responses in CSF/serum requires to be done as soon as possible.

Our study has several limitations as follows: (1) It is a pity that we neglected the electroencephalogram (EEG) examination. Although the diagnosis is based on clinical manifestations, neuroimaging examination and the test of autoantibodies in CSF/serum, EEG examinations often show abnormalities in 90% or even more patients with anti-NMDAR encephalitis, characterized by focal or generalized slow activity with or without epileptic discharges (7). Moreover, EEG recording can be normalized after effective treatment (2). We did not repeat the autoantibody test timely, resulting in a delayed diagnosis, for we neglected that the patient had received immunoregulatory therapy before the autoantibody test, which could result in a false-negative result from an autoantibody test (3). Six months follow-up is short relatively to assess the long-term outcome and frequency of relapses after the identification of concurrent antibodies. Fortunately, the patient had a good prognosis without any neurological sequels, and lesions in MRI had almost disappeared after mycophenolate mofetil (MMF) and corticosteroid treatment.

In summary, our case provides several clues that patients with recurrent CNS demyelination, particularly in the presence of brainstem lesion and cortical involvement on MRI, overlapping autoimmune diseases should be taken into consideration. Repeated autoantibody tests against AE, demyelinating disorders, and paraneoplastic syndrome are necessary for a definite and early diagnosis and therapy. Moreover, the diagnosis can be identified by a combination of other factors, including previous recurrent episodes of autoimmune demyelinating disorders, clinical or MRI characteristics, laboratory examination, and immunohistochemistry with patients' CSF/serum. In the meanwhile, this case reminded us that second-line immunotherapy, including MMF, rituximab (RTX), azathioprine (AZA), ciclosporin (CsA), and cyclophosphamide, is recommended for anti-NMDAR encephalitis particularly combining with recurrent demyelinating disorders despite a good response to the first-line treatment including steroids, IVIG, and plasma exchange, which have been proven to have a better prognosis and less recurrence compared with those who did not receive (3).

## Conclusion

Our clinical observation implies that auto-antibodies of AE and myelin in CSF/serum should be re-examined in patients with CNS demyelination relapse even though autoantibodies were negative in the first test. For patients who suffer from anti-NMDAR encephalitis combining with anti-MOG CNS demyelination, second-line immunoregulatory therapies which were confirmed to have a better prognosis in this case should be started as soon as possible when first-line treatment has been proven ineffective to prevent the relapse of disease.

## Data Availability Statement

The original contributions presented in the study are included in the article/Supplementary Material, further inquiries can be directed to the corresponding author/s.

## Ethics Statement

Written informed consent was obtained from the individual(s) for the publication of any potentially identifiable images or data included in this article.

## Author Contributions

YG: formal analysis. B-YR: writing -original draft and investigation. B-YR, JH, and Z-WL: methodology. YG, QW, Z-WL: project administration. B-YR and Z-WL: resources and writing -review, editing. JH: supervision. QW: validation. All authors contributed to the article and approved the submitted version.

## Conflict of Interest

The authors declare that the research was conducted in the absence of any commercial or financial relationships that could be construed as a potential conflict of interest.

## References

[1] Dalmau J Lancaster E Martinez-Hernandez E Rosenfeld MR Balice-Gordon R. Clinical experience and laboratory investigations in patients with anti-NMDAR encephalitis. Lancet Neurol. (2011) 10:63–74. 10.1016/S1474-4422(10)70253-2 21163445 PMC3158385

[2] Adachi H Ide Y Takahashi T Yoneda Y Kageyama Y. [Cerebral cortical encephalitis with anti-myelin oligodendrocyte glycoprotein (MOG) antibody]. Rinsho Shinkeigaku. (2018) 58:767–70. 10.5692/clinicalneurol.cn-001224 30487364

[3] Titulaer MJ McCracken L Gabilondo I Armangué T Glaser C Iizuka T. Treatment and prognostic factors for long-term outcome in patients with anti-NMDA receptor encephalitis: an observational cohort study. Lancet Neurol. (2013) 12:157–65. 10.1016/S1474-4422(12)70310-1 23290630 PMC3563251

[4] Titulaer MJ Höftberger R Iizuka T Leypoldt F McCracken L Cellucci T. Overlapping demyelinating syndromes and anti-N-methyl-D-aspartate receptor encephalitis. Ann Neurol. (2014) 75:411–28. 10.1002/ana.24117 24700511 PMC4016175

[5] Zhou L ZhangBao J Li H Li X Huang Y Wang M. Cerebral cortical encephalitis followed by recurrent CNS demyelination in a patient with concomitant anti-MOG and anti-NMDA receptor antibodies. Mult Scler Relat Disord. (2017) 18:90–2. 10.1016/j.msard.2017.09.023 29141829

[6] Bibic VC Brust TB Burton JM. Neuromyelitis optica spectrum disorder presenting with concurrent autoimmune diseases. Mult Scler Relat Disord. (2019) 28:125–8. 10.1016/j.msard.2018.12.028 30593981

[7] Liu CY Zhu J Zheng XY Ma C Wang X. Anti-N-Methyl-D-aspartate receptor encephalitis: a severe, potentially reversible autoimmune encephalitis. Mediators Inflamm. (2017) 2017:1–14. 10.1155/2017/6361479 28698711 PMC5494059

[8] Li C Xiao L Liu X Yang W Shen W Hu C. A functional role of NMDA receptor in regulating the differentiation of oligodendrocyte precursor cells and remyelination. Glia. (2013) 61:732–49. 10.1002/glia.22469 23440860

[9] van Coevorden-Hameete MH de Graaff E Titulaer MJ Hoogenraad CC Sillevis SPA. Molecular and cellular mechanisms underlying anti-neuronal antibody mediated disorders of the central nervous system. Autoimmun Rev. (2014) 13:299–312. 10.1016/j.autrev.2013.10.016 24225076

[10] Wegener-Panzer A Cleaveland R Wendel E Baumann M Bertolini A Häusler M. Clinical and imaging features of children with autoimmune encephalitis and MOG antibodies. Neurology. (2020) 7:1–10. 10.1212/NXI.0000000000000731 32358225 PMC7217659

[11] Martinez-Hernandez E Guasp M García-Serra A. Clinical significance of anti-NMDAR concurrent with glial or neuronal surface antibodies[J]. Neurology. (2020) 94:2302–2310. 10.1212/WNL.0000000000009239 32161029

