# Supplementary material for: Case Report: Anti-NMDAR Encephalitis With Anti-MOG CNS Demyelination After Recurrent CNS Demyelination
Source: Front Neurol. 2021 Feb 24;12:639265. doi: 10.3389/fneur.2021.639265 (PMC7943444; doi:10.3389/fneur.2021.639265)

**Supplementary picture legend**

Supplemental Figure 1: Brain MRI of symptom onset (abnormal signal in right medulla oblongata and pontine, the lesions are marked by red circle). Decreased T1 (A) signal abnormalities and increased T2 (B), FLAIR (C), DWI (C) and ADC (E) signal abnormalities involving the right brainstem are shown. Ring gadolinium (Gd) enhancement around the lesion of brainstem are show in axial review (F1), sagittal review (F2), coronal review (F3) in brain MRI.


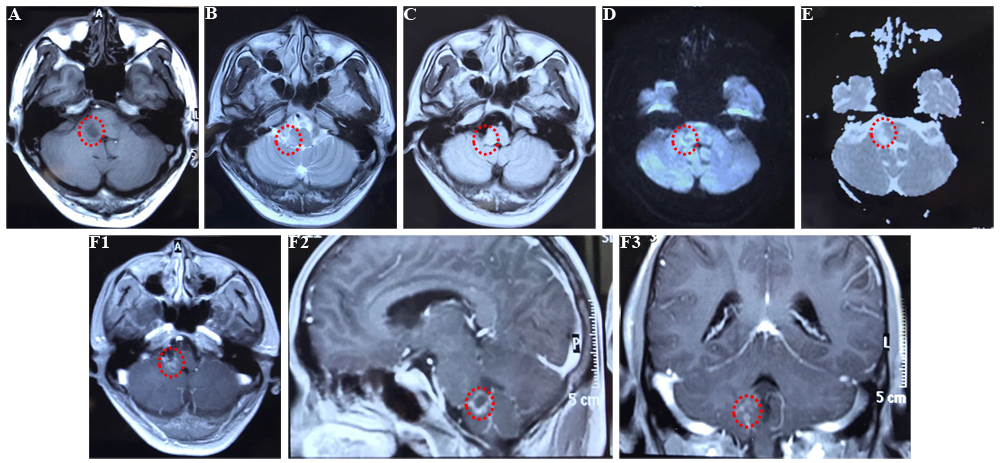


Supplemental Figure 2: Follow-up brain MRI after six months treatment with MMF and corticosteroids (The lesions in cerebral cortex and brainstem almost disappeared). (A1-F1) There are no obvious signal abnormalities in T1 (A1), T2 (B1), FLAIR (C1), DWI (D1), ADC (E1), and no obvious Gd enhancement (F1) at the brainstem level. (A2-F2) There are no obvious signal abnormalities in T1 (A2), T2 (B2), FLAIR (C2), DWI (D2) and ADC (E2) and no obvious Gd enhancement (F2) at the centrum semiovale level.


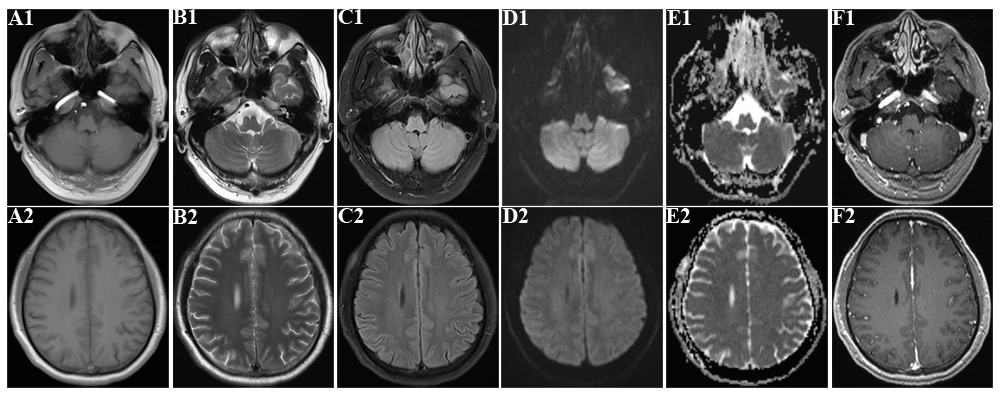

Supplement: Supplementary file 1 [file Table_1.DOC]
